# Pleural space infections after image-guided percutaneous drainage of infected intraabdominal fluid collections: a retrospective single institution analysis

**DOI:** 10.1186/s12893-015-0030-4

**Published:** 2015-04-11

**Authors:** Diego M Avella, Jennifer W Toth, Michael F Reed, Niraj J Gusani, Eric T Kimchi, Rickeshvar P Mahraj, Kevin F Staveley-O’Carroll, Jussuf T Kaifi

**Affiliations:** Department of Surgery, Pennsylvania State University College of Medicine, 500 University Drive, H070, Hershey, PA 17033 USA; Department of Medicine, Pennsylvania State University College of Medicine, 500 University Drive, H070, Hershey, PA 17033 USA; Department of Radiology, Penn State Milton S. Hershey Medical Center, Pennsylvania State University College of Medicine, 500 University Dr, Hershey, PA 17033 USA

**Keywords:** Pleural space infection, Pleural empyema, Percutaneous drainage, Intraabdominal abscess

## Abstract

**Background:**

Percutaneous drainage of infected intraabdominal fluid collections is preferred over surgical drainage due to lower morbidity and costs. However, it can be a challenging procedure and catheter insertion carries the potential to contaminate the pleural space from the abdomen. This retrospective analysis demonstrates the clinical and radiographic correlation between percutaneous drainage of infected intraabdominal collections and the development of iatrogenic pleural space infections.

**Methods:**

A retrospective single institution analysis of 550 consecutive percutaneous drainage procedures for intraabdominal fluid collections was performed over 24 months. Patient charts and imaging were reviewed with regard to pleural space infections that were attributed to percutaneous drain placements. Institutional review board approval was obtained for conduct of the study.

**Results:**

6/550 (1.1%) patients developed iatrogenic pleural space infections after percutaneous drainage of intraabdominal fluid collections. All 6 patients presented with respiratory symptoms and required pleural space drainage (either by needle aspiration or chest tube placement), 2 received intrapleural fibrinolytic therapy and 1 patient had to undergo surgical drainage. Pleural effusion cultures revealed same bacteria in both intraabdominal and pleural fluid in 3 (50%) cases. A video with a dynamic radiographic sequence demonstrating the contamination of the pleural space from percutaneous drainage of an infected intraabdominal collection is included.

**Conclusions:**

Iatrogenic pleural space infections after percutaneous drainage of intraabdominal fluid collections occur at a low incidence, but the pleural empyema can be progressive requiring prompt chest tube drainage, intrapleural fibrinolytic therapy or even surgery. Expertise in intraabdominal drain placements, awareness and early recognition of this complication is critical to minimize incidence, morbidity and mortality in these patients.

**Electronic supplementary material:**

The online version of this article (doi:10.1186/s12893-015-0030-4) contains supplementary material, which is available to authorized users.

## Background

Image-guided percutaneous drainage of intraabdominal fluid collections, commonly utilizing computer tomography (CT), ultrasound or fluoroscopy, is an effective and minimally invasive treatment method. It is widely preferred over surgical drainage due to lower overall complication rates, length of hospitalization and costs [1]. The success rate of image-guided drainage techniques is as high as 90% [1]. However, complications related to the percutaneous approach are underreported and need to be considered particularly when target collections are located in the upper abdomen and subphrenic spaces where the pleural space might get infected during catheter insertion or upsizing of existing drains. It can be a challenging procedure even for expert radiologists at high volume centers as correct drain placements are difficult and adjacent organ structures can be injured or perforated. Percutaneous drain placements in the subphrenic space carry the potential to penetrate the diaphragm and contaminate the pleural space with subsequent development of pleural space infections with rapid progression to complicated pleural effusions with loculations requiring chest tube or surgical drainage. Since overall mortality rate of pleural empyema ranges between 5% to 10%, prompt recognition and initiation of adequate therapy is essential [2].

This article presents a retrospective single institution analysis of 550 patients that underwent percutaneous drainage placements into the abdomen identifying 6 patients with pleural empyema illustrating the low incidence but aggressive clinical course of iatrogenic pleural space infections. They require prompt chest tube drainage and antibiotic coverage, but the contamination can rapidly progress to a complicated loculated pleural effusion or empyema requiring intrapleural fibrinolytic therapy or even surgical drainage with decortication. Significant morbidity and potentially even mortality can occur and immediate and adequate therapy has to be initiated.

## Methods

### Patients

This retrospective analysis reviewed 550 patients that underwent image-guided, percutaneous drainage placement for intraabdominal fluid collections over 24 months (July 2009 to June 2011) at Penn State Milton S. Hershey Medical Center, Pennsylvania State University. Medical records and imaging were reviewed and pleural space infections caused by intraabdominal drain placements were identified. Pleural space infections were classified according to the common criteria utilized to define pleural empyema that has been staged in three different phases by the *American Thoracic Society* (*ATS*) guidelines (exsudative (stage I), fibrinopurulent (stage II) and organization phase (stage III)) [3–5]. Approval of Penn State Milton S. Hershey Institutional review board (IRB) was obtained for this retrospective data analysis. Written informed consent was obtained from all 6 patients to include their clinical data in this study.

### Percutaneous image guided drainage of intraabdominal fluid collections

All procedures were performed under moderate sedation by a board-certified radiologist. Patients were placed in supine position on the CT scan or fluoroscopy table and images were obtained. The skin and subcutaneous tissues were anesthesized with local anesthesia. Subsequently a 19 gauge (G) coaxial needle was inserted under image-guidance and positioned with the tip located in the fluid collection. A multi-purpose drain, usually a 10 or 12 French pigtail catheter, was advanced into the fluid cavity over a wire. The *Cope* loop was formed, fluid was aspirated and samples forwarded for further laboratory and microbiological analysis. Final images confirmed appropriate positioning of the drain, and then the tubing was sutured in place and connected to a drainage bag.

### Intrapleural fibrinolytic therapy

In case of a complicated pleural effusion with loculations and septations, a mix of 10 mg of Tissue Plasminogen Activator (TPA), 5 mg of Recombinant Human Deoxyribonuclease I (Dornase alfa) plus 0.9% NaCl in a total volume of 50 ml were instilled through a chest tube. Upon infusion of the solution the chest tube was clamped for 1 hour followed by chest drain suction at −20 cm H2O. The instillation was repeated every 12 hours for 3 days. A pleural ultrasound, chest X-ray or CT scan was obtained 24 hours after completion of the last intrapleural fibrinolytic therapy [4].

## Results

In this retrospective single institution analysis over 24 months 550 patients underwent image-guided percutaneous intraabdominal drainage placement. Six (1.1%) patients developed a pleural space infection that all became symptomatic (e.g., pleuritic chest pain, dyspnea, tachypnea) and presented with signs of systemic infection (fever, leukocytosis) within a short period of time after drain placement (Table 1). Diagnosis of pleural space infection was established by the treating physicians between 24 hours to 17 days after drain placements. In all patients the treatment consisted of either continuation or initiation of systemic antibiotics and immediate chest tube drainage. Except for one patient (#1) who had an exsudative non-complicated pleural effusion (empyema stage I) all other patients were diagnosed with a complicated pleural effusion. Biochemical and cellular analysis were diagnostic for a pleural space infection and in addition in 3 (50%) out of 6 individuals bacteria isolated in the pleural effusions were identical to the ones in the abdomen collections. In 2 patients no bacteria were isolated in the pleural effusion, most likely to previous initiation of systemic and targeted antibiotic treatment for the intraabdominal infection. One patient had different bacteria isolated, however the pleural effusion culture was established two and a half weeks after intraabdominal drain placement that may have caused a shift and different selection in the bacterial types isolated.

**Table 1:** **Clinical characteristics and management of the six patients that developed iatrogenic pleural space infections after percutaneous drainage of intraabdominal/subphrenic infected collections**

**Patient**	**Age-gender**	**Primary pathology Primary intervention Indication for intraabdominal drainage**	**Time interval between percutaneous drainage and diagnosis of pleural space infection**	**Type of pleural space infection**	**Treatment of pleural space infection**	**Bacteria isolated in intraabdominal fluid collection and pleural effusion**	**Outcome**
**#1**	52-Female	Large retroperitoneal mass	24 hours after intraabdominal drain exchange	Uncomplicated pleural effusion exsudative phase of empyema (stage I)	Needle aspiration	Abdomen: *Candida glabrata* and *Staphyloccocus epidermidis*	Symptoms resolved within 24 hours after pleural drainage.
		Multivisceral *en bloc* resection (right upper abdominal quadrant)				Pleura: sterile	CT scan 10 days later demonstrated complete resolution.
		Subphrenic abscess					
**#2**	73-Female	Colon cancer metastatic to the liver	24 hours after additional intraabdominal drain placement	Complicated pleural effusion with loculations (fibrinopurulent phase of empyema (stage II))	Chest tube drainage	Abdomen: *Enterococcus sp.*, *Enterobacter cloacae and Lactobacillus sp.*	Symptoms resolved.
		Radiofrequency ablation of a liver metastasis				Pleura: sterile	Chest tube was removed 3 days later and patient discharged on day 8 after pleural drainage.
		Perihepatic abscess					Pleuritic pain resolved.
**#3**	51-Female	Gallbladder cancer Central hepatectomy with portal lymphadenectomy Perihepatic abscess	4 days after drain placement	Complicated pleural effusion (fibrinopurulent phase of empyema (stage II))	Chest tube drainage	Abdomen & Pleura: Methicillin-sensitive *Staphylococcus aureus* (MSSA)	Chest tube removed at day three. Patient discharged at day five post pleural drainage with complete recovery.
**#4**	77-Male	Colon cancer metastatic to the liver Partial hepatectomy Emphysematous cholecystitis (Video 1)	17 days after drain placement (cholecystostomy tube) (Video 1)	Complicated pleural effusion (fibrinopurulent phase of empyema (stage II))	Chest tube drainage & intrapleural fibrinolytic therapy	Abdomen: *Enterococcus faecalis, Enterobacter aerogenes* and *Escherichia coli*	Persistent biliary drainage 60 days after chest tube insertion that remained in place.
						Pleura (culture obtained 17 days after intraabdominal culture): *Pseudomonas aeruginosa*	Death related to primary pathology (75 days after liver surgery).
**#5**	57-Female	Alcohol-induced cirrhosis	10 days after additional (multiple) intraabdominal drain placements	Complicated pleural effusion (fibrinopurulent phase of empyema (stage II))	Chest tube drainage & intrapleural fibrinolytic therapy	Abdomen: *Enterococcus faecium*	Symptoms resolved. Chest tube removed 4
		Right lobe live donor liver transplantation. Subphrenic abscess				Pleura: *Enterococcus faecium*	days later. Complete recovery (last follow-up 1 year later).
**#6**	52-Male	Pancreatic tail cyst Distal splenopancreatectomy Peripancreatic abscess	36 hours after upsizing of drain (Figure 1)	Complicated pleural effusion (fibrinopurulent phase of empyema (stage II))	Chest tube drainage, VATS decortication	Abdomen & Pleura: *Pseudomonas aeruginosa*	Symptoms resolved. Patient discharged on day 8 after surgery. Complete recovery (last follow-up two years later).

Abbreviations: CT: Computer Tomography. VATS: Video-Assisted Thoracoscopic Surgery.

### Patient #1

A 52 year old female developed an infected right-sided subphrenic fluid collection after a multivisceral resection of a large retroperitoneal mass requiring percutaneous drainage with multiple catheter exchanges due to persistent and loculated fluid collections. *Candida glabrata* and *Staphyloccocus epidermidis* were isolated and treated for 6 weeks according to the antibiogram. Within 24 hours after an additional percutaneous intervention she developed severe respiratory distress secondary to a new large right-sided pleural effusion. An exsudative pleural effusion consistent with a pleural space infection in the biochemical analysis (pleural empyema stage I) was aspirated. However, no bacteria or fungal elements were isolated, most likely due to previous initiation of antibiotic therapy. Symptoms resolved within 24 hours and ten days after drainage a chest CT scan demonstrated complete resolution of the pleural effusion.

### Patient #2

A 73 year old female developed a liver abscess after radiofrequency ablation of a colorectal cancer metastasis. She underwent CT-guided percutaneous drainage of the intraabdominal collection and *Enterococcus sp.*, *Enterobacter cloacae and Lactobacillus sp.* were isolated and treated with systemic antibiotics. Percutaneous insertion of an additional intraabdominal drain was necessary due to incomplete drainage of the abscess. Within 24 hours after that procedure the patient developed progressive shortness of breath, tachypnea and tachycardia secondary to a new large right pleural effusion. Upon insertion of a 28 French chest tube, 250 ml of cloudy fluid were drained. Biochemical analysis and pleural ultrasound were consistent with a complicated pleural effusion. Cultures were sterile, most likely because of previous initiation of antibiotic therapy. The respiratory distress resolved after the insertion of the chest tube, in parallel to radiographic improvement. Specific antibiotic therapy targeting all bacteria isolated from the liver abscess was extended to 6 weeks, and the patient finally recovered completely.

### Patient #3

A 51 year old female underwent a central hepatectomy with portal lymphadenectomy for gallbladder adenocarcinoma. She developed a postoperative perihepatic abscess managed with CT-guided percutaneous placement of a 10 French pigtail drain. Methicillin-sensitive *Staphylococcus aureus* (MSSA) was isolated and treated with antibiotics for 6 weeks. Four days after the procedure she developed increasing right pleuritic pain caused by an infected large complicated pleural effusion that was treated with a chest tube draining 60 ml of purulent fluid. Microbiological cultures from the pleural fluid did also obtain MSSA. Despite chest tube placement the complicated pleural effusion was persistent and enlarging due to incomplete drainage caused by intrapleural loculations that were also visualized in a pleural ultrasound. Subsequently intrapleural fibrinolytic therapy was performed leading to resolution of the pleural effusion and symptoms. The chest tube was removed 4 days after insertion, and the patient was discharged home two days later with the abdominal drain in place that was removed 2 weeks after discharge. The patient was completely recovered in a six months clinical follow-up.

### Patient #4

A 77 year old male suffering from colon cancer metastatic to the liver underwent a partial hepatectomy that was complicated with a postoperative emphysematous cholecystitis treated with placement of a CT-guided percutaneous cholecystostomy drain. *Enterococcus faecalis, Enterobacter aerogenes* and *Escherichia coli* were isolated. Two and a half weeks after the insertion of the cholecystostomy tube, he developed right upper quadrant pain that prompted a cholangiogram through an intercostally placed cholecystostomy tube revealing a leak of injected contrast dye from the abdominal into the right pleural cavity (Additional file 1). A CT scan confirmed a right pleural effusion that was treated with insertion of a chest tube. *Pseudomonas aeruginosa* was isolated in the microbiological cultures. Due to persistent multiple loculations and septations in a residual complicated pleural effusion 48 hours after drainage, an intrapleural fibrinolytic therapy was performed that lead to complete resolution of the pleural effusion and to improvement of the respiratory symptoms. The chest tube was maintained due to persistent minimal amount of bilious fluid drainage. The patient was discharged to rehabilitation with the chest tube in place and a total course of 28 days of antibiotics. He remained free of respiratory symptoms and his abdominal pain and level of physical activity mildly improved over several weeks after the liver resection. However, the patient developed slowly progressing post-hepatectomy liver failure and died 75 days after liver surgery with both the chest and intraabdominal drains in place.

### Patient #5

A 57 year old female with a history of right lobe living donor liver transplantation complicated with multiple infected perihepatic bilious collections underwent repeated percutaneous drainage procedures. Ten days after the fourth percutaneous intraabdominal drain placement the patient developed right-sided pleuritic chest pain with a new right-sided pleural effusion. A chest tube initially drained a purulent pleural effusion, but after three days the chest tube output decreased and a highly loculated complicated pleural effusion was noted on ultrasound. An intrapleural fibrinolytic therapy was initiated and the patient improved clinically with resolution of the symptoms and pleural loculations on ultrasound. *Enterococcus faecium* was isolated from both the pleural fluid and intraabdominal biloma and antibiotic treatment was given for 6 weeks according to the antibiogram. The chest tube was removed 4 days after insertion. The patient finally recovered and was still doing well one year later in a routine clinical follow-up.

### Patient #6

**Figure 1:**
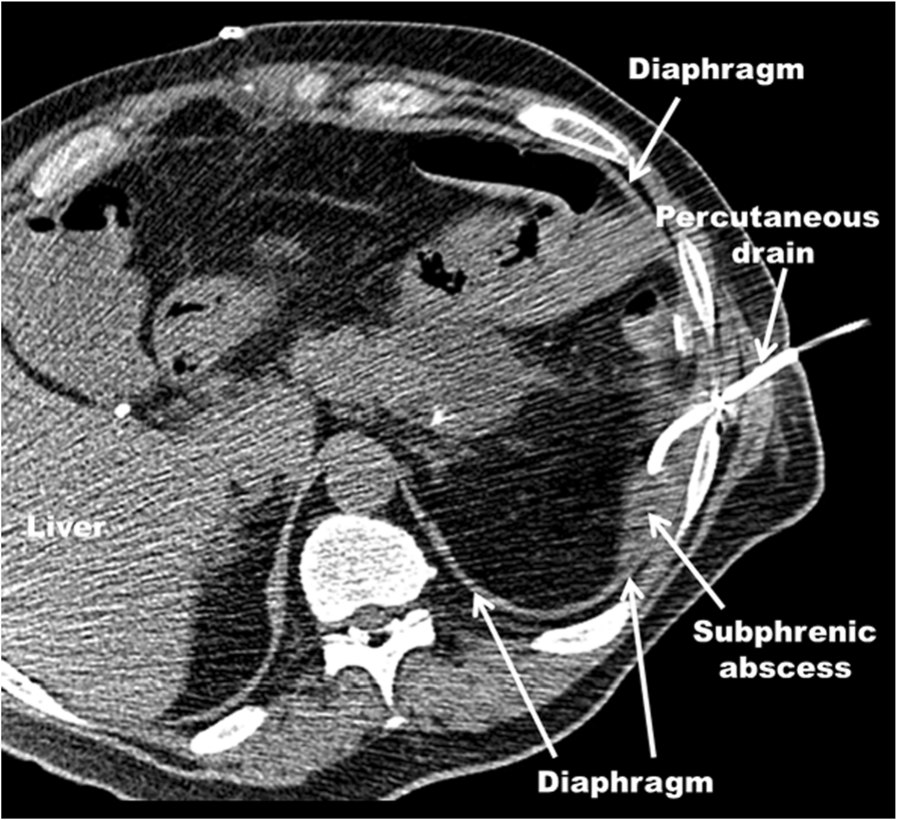
CT scan image illustrating a percutaneously placed intraabdominal catheter inserted through an intercostal space to drain an infected subphrenic fluid collection that occurred after a distal splenopancreatectomy (Patient #6). This intercostal drain had to be upsized from 12 to 14 French over a wire eight days after initial placement due to a persistent and enlarging subphrenic fluid collection. Subsequently the patient developed a symptomatic left-sided pleural space infection, most likely due to penetration of the diaphragm during upsizing of the drain. The pleural loculations and septations and the signs of systemic infected persisted despite antibiotics and catheter drainage and video-assisted thoracoscopic surgery (VATS) decortication was performed. Bacteria (*Pseudomonas aeruginosa*) isolated from the subdiaphragmatic collection and complicated pleural effusion were identical and treated accordingly with a 6 week course of antibiotics. The patient recovered quickly and was doing well in a three months clinical follow-up.

A 52 year old male underwent a distal splenopancreatectomy for a pancreatic tail cyst complicated with a left subphrenic abscess that was percutaneously drained through an intercostal space under CT-guidance (Figure 1). Systemic antibiotics were initiated, but despite drainage eight days later a persistent and enlarging peripancreatic abscess was seen on a follow-up CT scan. The well-positioned existing intraabdominal drain was upsized over a wire from a 12 to a 14 French catheter. Within the following 36 hours the patient developed tachypnea, tachycardia, hypoxia, fever and leukocytosis and a large left-sided complicated pleural effusion with loculations was detected in a chest CT scan and pleural ultrasound. A chest tube was inserted in the left pleural space draining 80 ml of purulent fluid. *Pseudomonas aeruginosa* bacteria were isolated from both the intraabdominal collection and pleural fluid and were treated accordingly with a 6 weeks course of systemic antibiotics. The pleural loculations and the signs of the systemic inflammatory response persisted despite antibiotics, catheter drainage and intrapleural fibrinolytic therapy. A video-assisted thoracoscopic surgery (VATS) debridement was subsequently indicated and performed. After an uneventful postoperative course the patient was discharged seven days later and was completely recovered in a three months outpatient clinical follow-up.

## Discussion

Percutaneous image-guided drainage of intraabdominal fluid collections is an effective, safe, low cost and morbidity procedure that decreases length of hospitalization in comparison to surgical drainage [1]. However, complications related to this minimally invasive percutaneous approach are underreported and need to be considered, particularly when infected target collections are located in the upper abdomen where adjacent organs can be injured. In general intraabdominal abscesses are a very frequent condition, but depending on the location of the fluid collection it is a challenge to establish adequate drainage for the treating physicians. In the present study the authors aimed to look at pleural space infections caused by diaphragmatic injury and bacterial pleural space contamination during insertion or upsizing of intraabdominal drains. This retrospective single institution analysis reviewed 550 patients that developed intraabdominal infected fluid collections after major visceral surgeries or as part of other intraabdominal disease processes. The overall incidence of iatrogenic pleural space infections after intraabdominal (including non-subphrenic locations) drain placements was low (1.1%). However the incidence after drain placements in exclusively subphrenic collections is probably higher, but our database did not differentiate between the specific locations of intraabdominal drain placements.

Standard diagnostic criteria for pleural empyema as outline in the criteria of the *American Thoracic Society* were applied, although the occurrence of an iatrogenic pleural space infection after drainage of infected intraabdominal collections obeys a different etiology and pathophysiology than a parapneumonic or postoperative pleural empyema [6, 7]. In our patients the spectrum of pleural space infection after percutaneous intraabdominal drainage procedures extended from exsudative pleural effusion (stage I) to complicated pleural effusion fibrinopurulent empyema (stage II) requiring aspiration or chest tube drainage, systemic antibiotics, intrapleural fibrinolytic therapy and in one case even a surgical intervention. Fortunately, upon diagnosis of the pleural empyema immediate therapy was initiated so that no one progressed to an organization phase of the empyema (stage III) that usually requires thoracotomy and open decortication of the lung to achieve lung expansion.

All affected six patients developed respiratory symptoms caused by the infected pleural effusions that were managed with needle aspiration or chest tube drainage in addition to systemic antibiotics. In three cases intrapleural fibrinolytic therapy had to be added because of loculations and one of them required surgical drainage due to persistent fibrinopurulent empyema (stage II) with persistent septations. In 5 (83%) out of 6 patients the treatment was successful, however in one patient who had a cholecystostomy tube placed for cholecystitis the intraabdominal and chest drains were left in place long-term due to persistent transdiaphragmatic biliary drainage, and eventually the patient died because of a chronic postoperative liver failure with a chronically drained but controlled intraabdominal and pleural space infection. A contrast dye injection into the intraabdominal cholecystostomy drain could fluoroscopically demonstrate the fistulous tract between the infected intraabdominal collection and the pleural space (Additional file 1). This illustrates that constant trafficking of contaminated material into the pleural space can occur after diaphragmatic perforation caused during intraabdominal drain placement.

For typically sterile drainage procedures of non-infected processes in the upper abdomen (e. g., liver biopsies, percutaneous transhepatic catheter (PTC) drainage placements for non-infected cholestasis or bilomas), the rate of pleural empyema has not been specifically addressed in studies about these procedures but is presumptively low [8]. However, in one retrospective study that reviewed patients with percutaneously drained pyogenic liver abscesses, 6.5% of them developed pleural effusions or empyema [1]. But the investigators did not focus on this specific complication and no demographic data or technical aspects of the procedures were provided.

The percutaneous approach for drainage of collections in the upper abdomen is limited by the presence of the ribcage. In two of our patients intercostal access that was chosen for drainage of infected collections may have caused pleural space infections. In contrast to the subcostal access in particular intercostal drain placements carry the potential of diaphragmatic perforation, pleural transgression and subsequent contamination of the pleural space. With regard to prevention of this complication, it is desirable to avoid transgression of the diaphragm with an oblique or an anterolateral approach with needle and drain insertion below the 10^th^ rib [9]. However, this may not always be feasible due to the subphrenic locations of the collections just underneath the diaphragmatic dome and the presence of other intraabdominal organs between the abdominal wall and the abscesses. In certain challenging cases a surgical (preferably minimally-invasive (laparoscopic)) but potentially safer intraabdominal drain placement under direct vision should be performed. More recently, transgastric drain placements under endoscopic ultrasound (EUS) guidance have also been described for successful management of postoperative intraabdominal fluid collections [10].

The initial indication for percutaneous drain placements of intraabdominal fluid collections should also be established with caution and preferably reviewed in an interdisciplinary setting. In fact, some studies have demonstrated no benefit of leaving a subphrenic drain in place over percutaneous needle aspiration alone [11]. Another approach to handle persistent intraabdominal fluid collections is upsizing the tubes to potentially achieve better drainage. However, a recent study demonstrated that upsizing the catheters did not increase the rate of successful drainage of intraabdominal collections, although catheters with a larger number of side holes may be advantageous [12]. In one of our patients, a pleural space infection occurred after the drain was upsized and that intervention contaminated the pleural space due to either guide wire manipulation or dilatation during catheter exchange. Of note is that in 3 other patients pleural space infections occurred after intraabdominal drain exchanges or placements of additional drains, indicating that multiple percutaneous interventions put patients at higher risk for an iatrogenic pleural space infection.

The challenge in recognition of this complication is that many patients develop sympathetic pleural effusions after abdominal surgeries that are reactive and generally not infected. In our patients the pleural effusions and space infections and symptom development were directly linked to the intraabdominal drain placements. Except for one patient all pleural effusions were complicated and the biochemical and cellular analysis was indicative for a pleural space infection, in addition to 3 (50%) out of 6 patients that had bacteria isolated in the pleural effusions that were identical to the ones in the abdomen. In 2 (33%) patients no bacteria were isolated in the pleural effusion, most likely to previous initiation of systemic and targeted antibiotic treatment for the intraabdominal infection. One patient had a different bacteria species isolated, however the pleural effusion culture was established two and a half weeks after bacterial culture from the abdomen that may have caused a shift and different selection in the bacterial species [13]. On retrospective review of imaging data diaphragmatic transgression was detected or determined to be highly likely. In addition a contrast dye extravasation into the pleural cavity demonstrating direct contamination is presented in a dynamic radiographic sequence (video). Pleural empyemas noted in our series were also not associated with pneumonias in any of the patients so that a parapneumonic pleural space infection was excluded.

## Conclusions

Iatrogenic pleural space infections after percutaneous drainage of infected intraabdominal fluid collections occur at a low incidence, but the development of a pleural empyema can be progressive requiring prompt chest tube drainage, intrapleural fibrinolytic therapy or even surgical decortication. Expertise in intraabdominal drain placements is critical to prevent iatrogenic pleural space infections. If a symptomatic, complicated, loculated and septated pleural effusion accompanied by systemic signs of infection develops shortly after catheter or needle insertion into an intraabdominal abscess, immediate drainage of the pleural space with adequate systemic antibiotic treatment is recommended under the assumption that pleural space contamination has occurred. Awareness of this potential complication permits earlier recognition and treatment to prevent development of a frank empyema occurring after percutaneous drainage of an infected intraabdominal fluid collection.

## Additional file

Additional file 1:
**Radiographic dynamic sequence demonstrating contrast dye contamination (cholangiogram) of the right pleural space after percutaneous drainage (cholecystostomy tube) of an infected intraabdominal collection (emphysematous cholecystitis) (Patient #4).** The video demonstrates the contrast injection into the drain with spillage into the right pleural space.
